# Assembly of an early-matured *japonica* (*Geng*) rice genome, Suijing18, based on PacBio and Illumina sequencing

**DOI:** 10.1038/sdata.2017.195

**Published:** 2017-12-19

**Authors:** Shou-Jun Nie, Yu-Qiang Liu, Chun-Chao Wang, Shi-Wei Gao, Tian-Tian Xu, Qing Liu, Hui-Lin Chang, Yu-Bao Chen, Peng-Cheng Yan, Wei Peng, Tian-Qing Zheng, Jian-Long Xu, Zhi-Kang Li

**Affiliations:** 1 Suihua Branch Institute, Heilongjiang Academy of Agricultural Sciences, 420 Gong-Nong West Road, Suihua, Heilongjiang 152000, China; 2 Institute of Crop Sciences/National Key Facility for Crop Gene Resources and Genetic Improvement, Chinese Academy of Agricultural Sciences, 12 South Zhong-Guan-Cun Street, Beijing 100081, China; 3 Beijing Computing Center, No. 7 Mid, Fengxian Rd. Yongfeng Industry Base, Beijing 100094, China; 4 Shenzhen Institute of Breeding for Innovation, Chinese Academy of Agricultural Sciences, Shenzhen 518120, China

**Keywords:** Structural variation, Plant breeding

## Abstract

The early-matured *japonica* (*Geng*) rice variety, Suijing18 (SJ18), carries multiple elite traits including durable blast resistance, good grain quality, and high yield. Using PacBio SMRT technology, we produced over 25 Gb of long-read sequencing raw data from SJ18 with a coverage of 62×. Using Illumina paired-end whole-genome shotgun sequencing technology, we generated 59 Gb of short-read sequencing data from SJ18 (23.6 Gb from a 200 bp library with a coverage of 59× and 35.4 Gb from an 800 bp library with a coverage of 88×). With these data, we assembled a single SJ18 genome and then generated a set of annotation data. These data sets can be used to test new programs for variation deep mining, and will provide new insights into the genome structure, function, and evolution of SJ18, and will provide essential support for biological research in general.

## Background & Summary

As the leading staple food resource for humans, rice has been adopted as an important model organism for biological research, especially for monocots. Asian cultivated rice (*Oryza sativa* L.) comprises two subspecies: *O. sativa* subsp. *japonica* (also known as *Keng*^1^ with the corresponding Pinyin, *Geng*) and subsp. *indica* (also known as *Hsien*^1^ with the corresponding Pinyin, *Xian*). Currently, *japonica*/*Geng*, especially the early-matured type, is becoming more and more important in rice production. In 2016, the cultivation area of early-mature *japonica*/*Geng* was more than 4 million ha in Northeast China.

It has become clear that one single genome is not enough to represent the huge amount of variation in rice genomes. Recently, in addition to the previously published *de novo* assemblies, including 93–11 (*indica*/*Xian*, two-line hybrid restorer), PA64S (admixture type with roughly 55% *indica*/*Xian*, 25% *japonica*/*Geng*, and 20% *javanica*, a two-line hybrid sterile line), IR64 (*indica*/*Xian*), DJ123 (*aus* type *indica*/*Xian*)^2^, HR-12 (*indica*/*Xian*)^3^, and Swarna (*indica*/*Xian*)^4^, three new sets of *indica*/*Xian* genomes have been released with the aid of third-generation sequencing technology for variation deep mining, including MH63RS1 (*indica*/*Xian*, three-line hybrid restorer), ZS97RS1 (*indica*/*Xian*, three-line hybrid maintainer)^5^, and R498 (*indica*/*Xian*, three-line hybrid restorer)^6^. These data sets have enriched our knowledge of the genomic variations of *indica*/*Xian* rice. Nevertheless, the genome of *japonica*/*Geng* is quite different from that of *indica*/*Xian*. Since the release of the gold standard genome of Nipponbare^7,8^, a medium-matured *japonica*/*Geng* variety with photosensitivity, the public availability of *japonica*/*Geng* genomes, especially for the early-mature type, remains largely blank.

According to our breeder’s experiences, early-matured *japonica*/*Geng* is a relatively unique type compared with the medium-matured *japonica*/*Geng*. In addition, common variations, such as single nucleotide polymorphisms (SNPs) within early-matured *japonica*/*Geng* group are relatively sparse. To improve the efficiency of molecular breeding in early-matured *japonica*/*Geng*, deep mining of further genome variations is urgently required. Short-read sequencing (SRS) technologies, such as Illumina HiSeq, have offered us an opportunity to access huge amounts of variations, including SNPs and short InDels, instantly from large sets of genomes^9^; however, to perform deeper mining of complex but critical variations, such as repeat sequence variations, long InDels, and structure variations (SVs), the technical bottleneck of the short sequencing read length remains a challenge. Currently, long-read sequencing (LRS) data are available with the aid of new technology, such as PacBio. However, the cost and error rate still remain relatively high. Thus, a scheme comprising LRS amended by SRS would represent a balanced choice for deep mining of genome variations^6,10^.

Early-mature *japonica*/*Geng* cultivar Suijing18 (SJ18) was newly developed by our joint project and was licensed for release in Northern China in 2014. It is a representative early-matured *japonica*/*Geng* cultivar harboring multiple elite traits (such as durable blast resistance, good grain quality, and high yield) and now represents more than 10% of the planting area of early-matured *japonica*/*Geng* in China. Therefore, we initiated a collaborative project to generate one high quality genome assembly for SJ18 to be used as a fundamental tool to help us investigate underlying genome variations in early-matured *japonica*/*Geng*. In this study, we report the resources and data sets that were generated and used for the deep mining of SJ18 genome variations: (1) raw PacBio LRS data, (2) Illumina whole-genome shotgun (WGS) SRS data, (3) the amended assembly of SJ18, (4) the annotation data based on the amended assembly of SJ18, and (5) the functional analysis results based on this annotation.

With the resources and data generated in this study, not only were we able assemble *de novo* a good quality genome sequence for early-matured *japonica*/*Geng*, but also were able to provide the scientific community with data to advance biological research at the genomic level, especially for the deep mining of genetic variations, and provided more information for genome-based molecular breeding of crops.

## Methods

### Plant material and library construction

**Figure 1:**
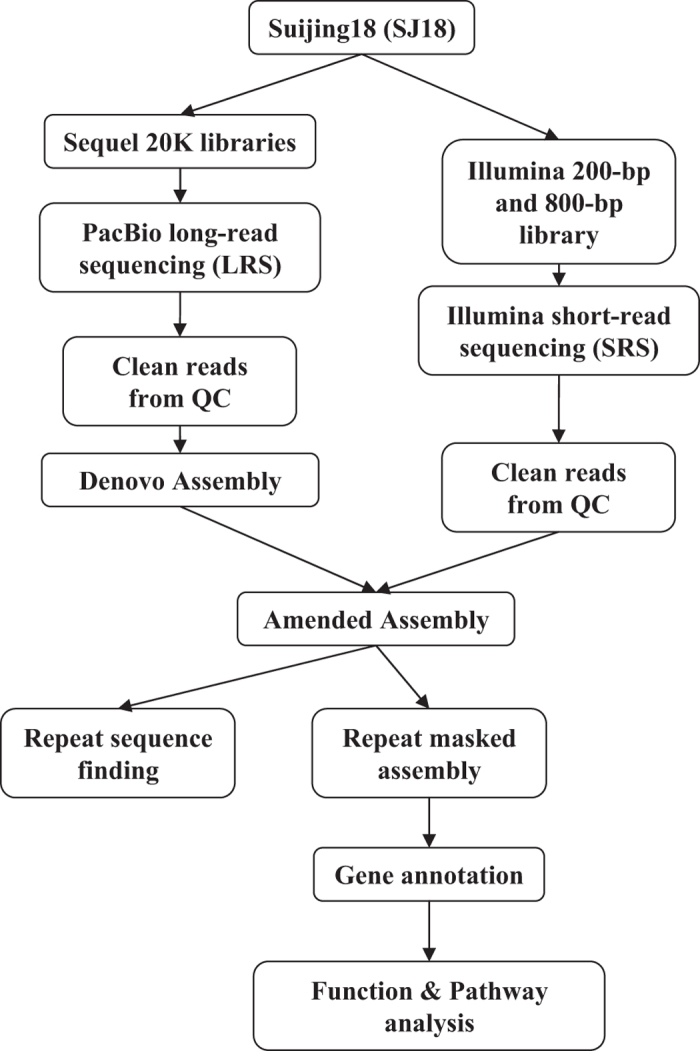
Outline of the workflow used to generate and analyze the genome data for Suijing18 (SJ18).

The early-matured *japonica*/*Geng* cultivar SJ18, which was developed by our own group, was licensed for release in 2014 and is now widely planted (more than 0.8 million hectare) in Heilongjiang province in Northeast China. High-molecular-weight genomic DNA was extracted from 10-day-old leaves of SJ18 (multiple seeds) using the modified CTAB method^11^, followed by 0.5× bead purification twice. The quality of the DNA sample was assessed using 0.75% agarose gel assays and Nanodrop (Nanodrop Technologies, Wilmington, DE, US), and was quantified using Qubit system (Thermo Fisher Scientific, Waltham, MA). The sample that met the quantity and quality standards was split into two parts, which were used to construct PacBio Sequel and Illumina libraries for LRS and SRS, respectively (Fig. 1).

The Sequel 20 K libraries were prepared using the standard protocol from PacBio and sequenced in the wet laboratory department of the Beijing Computing Center (http://www.bcc.ac.cn/) using a PacBio LRS instrument, model Sequel. The 200 bp- and 800 bp-libraries, with peak insert sizes of ~200 bp and ~800 bp, respectively, were prepared using an Illumina Truseq DNA library protocol (Illumina Kit FC-121-4001; Illumina Inc., San Diego, CA, USA). The qualities of libraries were checked using a standard protocol involving an Agilent 2,100 Bioanalyzer High Sensitivity Kit. After library profile analysis, the libraries were sequenced using 150 bp pair-end strategies with the Illumina HiSeq X10 platform (Illumina Inc.).

The amount of raw data from LRS was no less than 25 Gb, with a coverage of 62×. Using SRS, 59 Gb of raw data was generated, including 23.6 and 35.4 Gb of data from the 200 and 800 bp libraries, respectively. The total coverage of SRS was about 147×.

### Data analysis

The LRS data was screened and adjusted by the procedures embed in CANU^12^. Data that met the threshold of Q20 (corresponding to a 1% error rate) were adopted. *De novo* assembly was carried out for the LRS data using the CANU pipeline with default parameters, except for errorRate=0.045 and genomeSize=350 m. The SRS data were then aligned to the preliminary assembly using BWA^13^. In addition, the pilon package^14^ was adopted for the amendment process. The amended assembly represented the submitted version of the SJ18 sequence.

Based on the amended version of the SJ18 assembly, genome annotation was carried out using the following steps with default parameters, except for those indicated:

1. Tandem repeats were recognized by the TRF package^15^ with the following parameter settings: Match=2, Mismatch=7, Delta=7, PM=80, PI=10, Minscore=50, MaxPeriod=2,000. Other types of repeat sequences were recognized by RepeatModeler (http://www.repeatmasker.org/RepeatModeler/) with default settings. The database adopted for RepeatModeler analysis was an integrated library comprising Repbase^16^ (updated in January 2017), Dfam2 (ref. 17), and publicly available libraries containing *de novo* information for rice.
2. Annotation for non-coding RNA (ncRNA) was carried out by using cmsearch in Infernal^18^, searching the Rfam database V12.2 (http://rfam.xfam.org/) with a parameter setting of ‘—cyk -T10’ for microRNAs (miRNAs), small nuclear ribonucleic acid (snRNA), and small RNA (sRNA). The transfer RNA (tRNA) annotation was carried out using tRNAscan-SE^19^ with default settings.
3. We masked the repeats using RepeatMask with the parameter setting of ‘-nolow -no_is -norna’ and then annotated the SJ18 genome using multiple tools, including GENEID^20^ with parameter settings for rice, GeneMark^21^ with a setting of —ES —cores 24 —min_contig 100, SNAP^22^ with default setting, and AUGUSTUS^23^ with -species=rice. We compared the coding sequences (CDSs) and protein sequences from other rice genomes using PASA^24^ with default settings and GeneWise^25^ with a setting of ‘splice_gtag -sum -gff -quiet’. All the annotation results were integrated and screened using EVidenceModeler (EVM)^26^.
4. The predicted coding genes from SJ18 were translated into protein sequences and aligned to the proteins from plant species in the Uniprot database (http://www.uniprot.org/) and Kyoto Encyclopedia of Genes and Genomes (KEGG) database (http://www.genome.jp/kegg/), respectively, using BLASTP. The threshold was set to e-value<1e-8, and the best hits were submitted for further analysis.
5. Gene ontology (GO) analysis was carried out based on the above functional annotation results by using topGO^27^. The biological process (BP), cellular component (CC), and molecular function (MF) matches were listed. The secondary binding point was chosen in the analysis. The annotated proteins from SJ18 were submitted for pathway analysis using KEGG.

## Data Records

Raw PacBio long-read sequencing (LRS) data are available through the NCBI SRA with the accession number SRR5877285 (Data Citation 1). All Illumina short-read sequencing (SRS) data for SJ18 can be found at the NCBI SRA with accession numbers SRR5880534 (Data Citation 2) and SRR5880533 (Data Citation 3). The assembled SJ18 genome version 1 is available at the NCBI with the accession number PDFQ00000000 (Data Citation 4). All these raw data are also available at figshare (Data Citation 5). The analyzed data are available at figshare (Data Citation 5) or through the URLs offered by figshare and the Rice Functional Genomics and Breeding (RFGB) database^28^ (Table 1).

## Technical Validation

**Figure 2:**
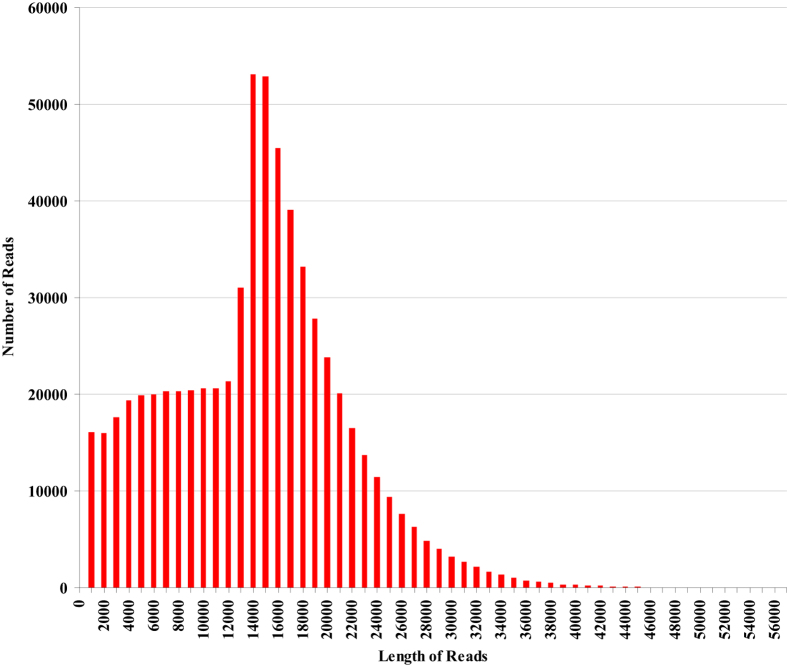
Distribution of high quality reads from PacBio long-read sequencing (LRS) for Suijing18 (SJ18).

The LRS data were screened and amended with the SRS data using the CANU package with default settings. Possible sequencing errors were further minimized by removing reads that aligned with high scores to the downloaded sequences from bacteria, fungi, or human genomes from GenBank using BWA. Finally, a total of 648,237 high-quality LRS reads that passed this quality check step were submitted for assembly. The distribution of these reads is shown in Fig. 2.

The raw SRS data was screened using the Trimmomatic package^29^, which removed the adaptors and the reads with a quality value lower than 20 (corresponding to a 1% error rate).

We also compared the parameters of SJ18 with other assemblies. The statistics of the assembled contigs are shown in Table 2. The statistics of repeat sequences are shown in Table 3 in comparison with Nipponbare (medium-matured *japonica*/*Geng*) and R498 (*indica*/*Xian*, the most recently available rice assembly).

## Additional information

**How to cite this article:** Nie, S.-J. *et al.* Assembly of an early-matured *japonica* (*Geng*) rice genome, Suijing18, based on PacBio and Illumina sequencing. *Sci. Data* 4:170195 doi: 10.1038/sdata.2017.195 (2017).

**Publisher’s note:** Springer Nature remains neutral with regard to jurisdictional claims in published maps and institutional affiliations.

## Supplementary Material



## Figures and Tables

**Table 1:** Analyzed data resources for Suijing18 (SJ18) deposited at figshare or the Rice Functional Genomics and Breeding (RFGB) database.

**Subjects**	**Title**	**Public links**
*De novo* assembly	Suijing18 Denovo assembly version 1	Data Citation 5 or http://www.rmbreeding.cn/downloads/sj18/SJ18_v1.fasta.gz
Repeat-masked *de novo* assembly	Repeat masked data based on Suijing18 Denovo assembly version 1	Data Citation 5 or http://www.rmbreeding.cn/downloads/sj18/SJ18_v1.masked.fasta.gz
Gene annotation results	Annotated genes based on Suijing18 Denovo assembly version 1	Data Citation 5 or http://www.rmbreeding.cn/downloads/sj18/SJ18.gene.gff3.gz
ncRNA annotated	Annotated ncRNAs based on Suijing18 Denovo assembly version 1	Data Citation 5 or http://www.rmbreeding.cn/downloads/sj18/SJ18.ncRNA.gff3.gz
Repeats annotated	Annotated Repeats based on Suijing18 Denovo assembly version 1	Data Citation 5 or http://www.rmbreeding.cn/downloads/sj18/SJ18.repeat.gff3.gz
Functional annotation results based on the alignments from KEGG	Annotated proteins based on Suijing18 Denovo assembly version 1 and KEGG database	Data Citation 5 or http://www.rmbreeding.cn/downloads/sj18/SJ18.kegg.xls.gz
Functional annotation results based on the alignments from UniProt	Annotated proteins based on Suijing18 Denovo assembly version 1 and Uniprot database	Data Citation 5 or http://www.rmbreeding.cn/downloads/sj18/SJ18.uniprot.xls.gz
Pathway analysis results	Gene ontology analysis results based on Suijing18 Denovo assembly version 1	Data Citation 5 or http://www.rmbreeding.cn/downloads/sj18/SJ18.pathway.zip
KEGG, Kyoto Encyclopedia of genes and genomes; ncRNA, non-coding RNA.		

**Table 2:** Comparisons between Suijing18 (SJ18) and the other datasets for representative assembled contigs publicly available and the annotated ncRNAs.

***Subspecies***	**SJ18 Early-matured** ***japonica*****/** ***Geng***	**IRGSP1.0 Medium-matured** ***japonica*****/** ***Geng***	**R498** ***Indica*****/*****Xian*** **Three-line hybrid restorer**	**ZS97RS1** ***Indica*****/*****Xian*** **Three-line hybrid maintainer**	**MH63RS1** ***Indica*****/*****Xian*** **Three-line hybrid restorer**	**HR-12** ***Indica*****/*****Xian***	**9,311** ***Indica*****/*****Xian*** **Two-line hybrid restorer**	**PA64S** ***Indica*****/** ***Xian*** **Two-line hybrid sterile line**	**IR64** ***Indica*****/*****Xian***	**DJ123** ***Aus*** **type of** ***indica*****/*****Xian***
Total nucleotides (Mb)	418.9	373.2	390.3–423.2	346.9	359.9	389.8	374.6–466.0	382.0	316.3	321.2
N50 contig length (bp)	2,467,626	7,711,345	1,185,206	2,339,070	3,097,358	28,500	6,690	17,000	22,200	25,500
Total genes	38,456	39,045	38,714	34,610	37,324	56,284	40,745	37,162	37,768	37,812
tRNA	434	244	NA	592	589	NA	734–993	NA	NA	NA
snoRNA	681	NA	NA	449	457	NA	NA	NA	NA	NA
snRNA	108	NA	NA	92	97	NA	3,374	NA	NA	NA
rRNA	88	724	NA	40	60	NA	752	NA	NA	NA
miRNA	173	146	NA	341	363	NA	3,806	1,155	NA	NA
tRNA, transfer RNA; snoRNA, small nucleolar RNA; snRNA, small ribonuclear RNA; rRNA, ribosomal RNA; miRNA, microRNA.										

**Table 3:** Different types of repeat sequences found in the Suijing18 (SJ18) assembly (version 1).

**Repeat_Type**	**SJ18**		**Nipponbare**		**R498**	
	**Length (bp)**	**%**	**Length (bp)**	**%**	**Length (bp)**	**%**
Class I: Retrotransposon	94,087,436	22.3	105,098,791	28.2	117,509,061	30.1
LTR-Retrotransposon	88,392,161	21.0	98,903,987	26.5	111,173,484	28.4
LTR/Gypsy	72,477,718	17.2	65,915,787	17.7	80,330,011	20.6
LTR/Copia	14,161,220	3.4	17,931,866	4.8	15,079,454	3.9
LTR/Other	1,753,223	0.4	15,056,334	4.0	15,764,019	4.0
Non-LTR Retrotransposon	5,695,275	1.4	6,194,804	1.7	6,335,577	1.6
SINE	362,005	0.1	796,311	0.2	848,061	0.2
LINE	5,333,270	1.3	5,398,493	1.5	5,487,516	1.4
Class II: DNA Transposon	69,249,868	16.4	40,716,340	10.9	40,743,536	10.4
EnSpm/CACTA	10,690,200	2.5	14,117,095	3.8	13,264,041	3.4
hAT	5,494,725	1.3	1,641,580	0.4	1,897,505	0.5
Harbinger	9,746,844	2.3	3,729,352	1.0	3,882,866	1.0
Tc1/Mariner	6,525,750	1.6	462,697	0.1	607,903	0.2
MuDR	16,081,157	3.8	5,872,349	1.6	5,993,554	1.5
Helitron	12,538,290	3.0	1,850,232	0.5	1,702,664	0.4
Other	8,172,902	1.9	13,043,035	3.5	13,395,003	3.4
Other tandem repeat	18,811,934	4.5	3,935,022	1.1	4,548,484	1.2
Low Complexity	313,050	0.1	22,478	0.0	17,672	0.0
Unclassified	13,830,076	3.3	1,117,819	0.3	1,607,036	0.4
Total	196,292,364	46.5	150,890,450	40.4	164,425,789	42.1
LTR, Long Terminal Repeats; SINE, Short Interspersed Nuclear Element; LINE, Long Interspersed Nuclear Element; EnSpm, Enhancer/Suppressor mutator; hAT, hobo-Ac-Tam3; MuDR, MuDR: A generic notation for a Mu transposon containing a sequence necessary to permit Mu transposition and related behaviors. The ‘DR’ is in honor of Dr Donald S. Robertson, who discovered and characterized the original Mutator lines.						

## References

[d1] NCBI Sequence Read Archive 2017 SRP113746

[d2] NCBI Sequence Read Archive 2017 SRP113817

[d3] NCBI Sequence Read Archive 2017 SRP113816

[d4] NCBI Assembly 2017 GCA_002573525

[d5] Figshare Zheng T. Q. 2017 https://doi.org/10.6084/m9.figshare.c.3835939

